# From molecular dynamics to Brownian dynamics

**DOI:** 10.1098/rspa.2014.0036

**Published:** 2014-07-08

**Authors:** Radek Erban

**Affiliations:** Mathematical Institute, University of Oxford, Radcliffe Observatory Quarter, Woodstock Road, Oxford OX2 6GG, UK

**Keywords:** multi-scale modelling, molecular dynamics, Brownian dynamics

## Abstract

Three coarse-grained molecular dynamics (MD) models are investigated with the aim of developing and analysing multi-scale methods which use MD simulations in parts of the computational domain and (less detailed) Brownian dynamics (BD) simulations in the remainder of the domain. The first MD model is formulated in one spatial dimension. It is based on elastic collisions of heavy molecules (e.g. proteins) with light point particles (e.g. water molecules). Two three-dimensional MD models are then investigated. The obtained results are applied to a simplified model of protein binding to receptors on the cellular membrane. It is shown that modern BD simulators of intracellular processes can be used in the bulk and accurately coupled with a (more detailed) MD model of protein binding which is used close to the membrane.

## 1. Introduction

Brownian dynamics (BD) simulations have been used for the modelling of a number of spatio-temporal processes in cellular and molecular biology in recent years, including models of intracellular calcium dynamics [1], the MAPK pathway [2] and signal trasduction in *Escherichia coli* chemotaxis [3]. In these applications, trajectories and interactions between key biomolecules (e.g. proteins) are calculated using BD methods, while other components of the system (e.g. solvent molecules), which are of no special interest to a modeller, are not explicitly included in the simulation, but contribute to the dynamics of Brownian particles collectively as a random force. This reduces the dimensionality of the problem, making BD less computationally intensive than the corresponding molecular dynamics (MD) simulations.

Denoting the position of a Brownian particle by **X**=[*X*_1_,*X*_2_,*X*_3_] and its diffusion constant by *D*, a simple model of Brownian motion is given by the (overdamped) Langevin equation

1.1 $$\text{d}X_{i}=\sqrt{2D}\;\text{d}W_{i},\;i=1,2,3,$$

where *W*_*i*_, *i*=1,2,3, are three independent Wiener processes [4]. BD approaches which are based on (1.1) have been implemented in a number of software packages designed for spatio-temporal modelling in systems biology, including Smoldyn [5], MCell [6], Green's function reaction dynamics [7] and the first-passage kinetic Monte Carlo method [8]. The software package Smoldyn discretizes (1.1) using a fixed time step Δ*t*, i.e. it computes the time evolution of the position **X**≡**X**(*t*) of each molecule by

1.2 $$X_{i}(t+\Delta t)=X_{i}(t)+\sqrt{2D\Delta t}\;\xi_{i},\;i=1,2,3,$$

where [ξ_1_,ξ_2_,ξ_3_] is a vector of normally distributed random numbers with zero mean and unit variance. A different BD approach is implemented in the Green's function reaction dynamics [2] which evolves time using a variable time step. It approximately computes the time when the next reactive event happens. This means that trajectories of molecules which are not surrounded by other reactants can be simulated over longer time steps.

Although the BD models are becoming a popular choice for stochastic modelling of intracellular spatio-temporal processes, several difficulties prevent the use of BD for some systems. First of all, detailed BD models are often more computationally intensive than coarser spatio-temporal models which are written for concentrations of biochemical species. In some applications (e.g. intracellular calcium dynamics [1] or actin dynamics in filopodia [9]), individual trajectories (computed by BD) are important only in certain parts of the computational domain, while in the remainder of the domain a coarser, less detailed, method can be used. In these applications, the computational intensity of BD simulations can be decreased by using multi-scale methods which efficiently and accurately combine models with a different level of detail in different parts of the computational domain [10,11].

Another difficulty of BD simulations in cell and molecular biology is that detailed BD models require more parameters than coarser (macroscopic) models. In some studies, macroscopic parameters are used to infer BD parameters [12,5]. For example, knowing the macroscopic reaction rate *k* of a bimolecular reaction *A*+*B*→*C* and diffusion constants of reactants, one can calculate a (microscopic) reaction radius of BD simulations which gives the corresponding macroscopic parameters in the limit of many particles. In the classical Smoluchowski limit [13], a bimolecular reaction occurs whenever the distance of reactants is less than the reaction radius

1.3 $$\varrho =\frac{k}{4\pi (D_{A}+D_{B})},$$

where *D*_A_ (resp. *D*_B_) is the diffusion constant of reactant A (resp. B). Although this approach is commonly applied in stochastic reaction–diffusion models, it is not the most satisfactory, because different microscopic models can lead to the same macroscopic process and parameters [14,12]. For example, the simplest Smoluchowski model (1.3) assumes that all collisions are reactive but, in reality, many non-reactive collisions of molecules happen before a reactive collision occurs. Therefore, some algorithms postulate that molecules only react with a certain rate (probability) when the distance between reactants is less than a modified reaction radius (which is larger than *ϱ*). Other methods discretize the Langevin equation with time step Δ*t* and substitute the Smoluchowski formula (1.3) (which is valid for an infinitely small time step) by a tabulated function computed numerically [5]. However, all of these approaches are verified by considering the macroscopic limit (of many reactants) and showing that the reaction occurs with the given rate *k* in this limit.

A different approach to parametrize BD models is to use a more detailed description written in terms of MD. In this paper, we investigate connections between BD and MD models with the aim of developing and analysing multi-scale methods which couple BD and MD simulations. We consider a (computationally intensive) MD simulation in a domain *Ω* which is either one or three dimensional, i.e. Ω⊂R or $\Omega \subset R^{3}$. Our main goal is to design and analyse multi-scale methods which can compute spatio-temporal statistics with an MD level of detail in the subdomain *Ω*_D_⊂*Ω*. We define

1.4 $$\Omega_{C}=\Omega \setminus \bar{\Omega_{D}}\;\mathrm{and}\;I=\partial \Omega_{D}\cap \partial \Omega_{C},$$

where *Ω*_D_ and *Ω* are open sets, the (open) set *Ω*_C_ is the complement of *Ω*_D_ and *I* is the shared interface (boundary) between *Ω*_D_ and *Ω*_C_. In the multi-scale set-up (1.4), we use a detailed MD model in *Ω*_D_ and a coarser BD model in *Ω*_C_.

In this paper, we focus on a simple MD approach which is introduced in §2 and §3. A few (heavy) particles with mass *M* and radius *R* are coupled with a large number of light point particles with masses *m*≪*M*. The collisions of particles are without friction, which means that post-collision velocities can be computed using the conservation of momentum and energy [15,16]. We will introduce and study three MD models which make use of elastic collisions. They will be denoted as MD models [A], [B] and [C] in what follows. More complicated MD approaches are discussed in §7.

The first MD model [A] is introduced in §2. It is a one-dimensional MD model where all particles move along the real line. In particular, the radius *R* does not have to be considered, because it has no influence on the dynamics of large particles. In one dimension, heat bath particles cannot pass each other, which makes the MD model [A] different from three-dimensional models in §3 where heat bath particles (points) do not interact with each other.

In §3, we introduce two three-dimensional models, denoted [B] and [C], where the non-zero radius *R* is one of the key parameters. To make one- and three-dimensional models comparable, we keep *R* fixed in the three-dimensional model and we study the behaviour of all MD models in the limit M/m→∞. This limit can be achieved in many different ways. For example, we can keep *m* fixed and pass M→∞, or we can keep *M* fixed and pass *m*→0. In what follows, we define the parameter

1.5 $$\mu =\frac{M}{m}.$$

This parameter is dimensionless, even if we assume that *M* and *m* have physical units of mass. However, in this paper, all parameters are considered dimensionless for simplicity. We are interested in the limit μ→∞.

All three models [A], [B] and [C] converge in appropriate limits to the Brownian motion of large particles given by (1.1). One can also show that these models converge to the Langevin description [15–17]

1.6 $$\begin{array}{rl} \text{d}X_{i} & =V_{i}\;\text{d}t, \end{array}$$

1.7 $$\begin{array}{rl} \text{d}V_{i} & =-\gamma V_{i}\;\text{d}t+\gamma \sqrt{2D}\;\text{d}W_{i},\;i=1,2,3, \end{array}$$

where [*X*_1_,*X*_2_,*X*_3_] is the position of a diffusing molecule, [*V*_1_,*V*_2_,*V*_3_] is its velocity, *D* is the diffusion coefficient and *γ* is the friction coefficient. This description can be further reduced to (1.1) in the overdamped limit γ→∞. We overview the results which relate MD models [A], [B] and [C] with Brownian motion in §2 and §3.

Both (1.1) or (1.6)–(1.7) reduce the dimensionality of the problem, making BD less computationally intensive than the corresponding MD simulations. In §4 and §5, we study how MD models [A], [B] and [C] can be used in one part *Ω*_D_ of the computational domain *Ω* and the BD models (1.1) or (1.6)–(1.7) in the remainder *Ω*_C_, making use of the notation (1.4). We apply our findings to a simplified model of protein binding to receptors in §6. We conclude with discussing our results in §7.

## 2. One-dimensional molecular dynamics model [A]

The MD model [A] is described in terms of positions *x*^*i*^ and velocities *v*^*i*^, *i*=1,2,3,…, of heat bath particles, and positions *X*^*i*^ and velocities *V* ^*i*^, *i*=1,2,…,*N*, of heavy particles of mass *M*≫*m*, where *m* is the mass of a heat bath particle [15]. In our computer implementations, we will consider a finite number of heat bath particles. However, we formulate the MD model in terms of (countably) infinitely many heat bath particles which are initially distributed along the real line according to the Poisson distribution with density

2.1 $$\lambda_{\mu }=\frac{1}{4}\sqrt{\frac{\pi (\mu +1)\gamma }{2D}},$$

where *μ* is given by (1.5), and *D* and *γ* are positive constants. This means that the probability that there are *j* particles in a subinterval [a,b]⊂R, *a*<*b*, is equal to

$$\frac{{\left(\lambda_{\mu }(b-a)\right)}^{j}}{j!}\exp[-\lambda_{\mu }(b-a)],$$

where (*b*−*a*) is the length of the interval [*a*,*b*]. Initial velocities of heat bath particles are given by the normal distribution

2.2 $$f_{\mu }(v)=\frac{1}{\sigma_{\mu }\sqrt{2\pi }}\exp\left(-\frac{v^{2}}{2\sigma_{\mu }^{2}}\right),\;\text{where}\;\sigma_{\mu }=\sqrt{(\mu +1)D\gamma }.$$

Let us consider a model with a single heavy particle, i.e. *N*=1. Then its location *X*^1^ and velocity *V* ^1^ will be denoted as *X* and *V* to simplify our notation. Whenever the heavy particle collides with the light particle with velocity *v*^*i*^, their velocities are updated using the conservation of mass and momentum by

2.3 $$\tilde{V\;}\;\;=\frac{M-m}{M+m}V+\frac{2m}{M+m}v^{i}$$

and

2.4 $${\tilde{v}}^{i}=\frac{m-M}{M+m}v^{i}+\frac{2M}{M+m}V,$$

where tildes denote post-collision velocities. Using (1.5), the equations (2.3) and (2.4) can be rewritten as

2.5 $$\tilde{V\;}\;\;=\frac{\mu -1}{\mu +1}V+\frac{2}{\mu +1}v^{i}\;\mathrm{and}\;{\tilde{v}}^{i}=\frac{1-\mu }{\mu +1}v^{i}+\frac{2\mu }{\mu +1}V.$$

The following result can be shown for the above MD model [A].

**Lemma 2.1**

*Let* *γ*>0 *and* *D*>0. *Let us consider the heavy particle of mass* *M* *with initial position* *X*_*μ*_(0)=*X*_0_ *and initial velocity* *V*_*μ*_(0)=*V*_0_ *which is subject to elastic collisions* *(2.5)* *with heat bath particles of mass* *m* *whose initial positions and velocities are distributed according to* *(2.1)* *and* *(2.2)*. *Then* *X*_*μ*_ *and* *V*_*μ*_ *converges* (*as* μ→∞) *in distribution to the solution* *X* *and* *V* *of equations*

2.6 $$dX=V\;dt\;\text{and}\;dV=-\gamma V+\gamma \sqrt{2D}\;dW,$$

*where* *X*(0)=*X*_0_ *and* *V* (0)=*V*_0_. *That is*, *X* *and* *V* *solve the one-dimensional version of equations* *(1.6)* *and* *(1.7)*.

**Proof.**

This lemma can be proved using the main theorem in Holley [15], where it is shown that a similar process converges to the Ornstein–Uhlenbeck process (1.7) for velocities. Although our function *f*_*μ*_ does not satisfy all assumptions of the main theorem of Holley [15], a simple rescaling of our parameters leads to a process which is covered by Holley's theorem. In §4 of this paper, we also rederive this result as one of the consequences of multi-scale analysis (see (4.11)). □

As the goal of this paper is to study the behaviour of computational algorithms, we formulate the MD model [A] in a finite domain [−*L*,*L*], i.e. we consider a finite number *n*≡*n*(*t*) of heat bath particles which are at positions *x*^*i*^∈[−*L*,*L*] with velocities $v^{i}\in (-\infty ,\infty )$, *i*=1,2,…,*n*. We want to formulate boundary conditions of our problem so that the spatio-temporal statistics in [−*L*,*L*] are equivalent to spatio-temporal statistics of the original unbounded process. The following lemma will be useful for designing appropriate boundary conditions.

**Lemma 2.2**

*Let* b∈R *and* Δ*t*>0. *Let us assume that heat bath particles are distributed according to the Poisson distribution with density* *(2.1)* *in the interval* (−∞,b). *Their initial velocities are given according to* *(2.2)* *and there are no particles in the interval* (b,∞) *at time* *t*=0. *Then the average number of particles in the interval* (b,∞) *at time* *t*=Δ*t* *is*

2.7 $$\frac{\gamma (\mu +1)\Delta t}{8}.$$

*The positions* *x* *and velocities* *v* *of these particles are distributed according to*

2.8 $$H(b-x+v\Delta t)\lambda_{\mu }f_{\mu }(v),$$

*where* *H*(⋅) *is the Heaviside step function. In particular, the positions of the particles at point* x∈(b,∞) *are distributed at time* *t*=Δ*t* *according to*

2.9 $$\varrho (x;\Delta t,b)\equiv \frac{\lambda_{\mu }}{2}\;\mathrm{erfc}\left(\frac{x-b}{\sigma_{\mu }\Delta t\sqrt{2}}\right),\;\text{for}\;x\in (b,\infty ),$$

*where* $\mathrm{erfc}(z)=2/\sqrt{\pi }\int_{z}^{\infty }\exp(-s^{2})\;ds$ is the complementary error function.

**Proof.**

Particles which are at point x∈(b,∞) at time *t*=Δ*t* were previously at point *x*−*vΔt* at time *t*=0. In particular, there will be non-zero heat bath particles with velocity *v* at point *x* at time *t*=Δ*t* provided that *x*−*v*Δ*t*<*b*, which implies (2.8). Consequently, the density of particles which are at point x∈(b,∞) at time *t*=Δ*t* is

$$\begin{array}{rl} \varrho (x;\Delta t,b) & =\int_{-\infty }^{\infty }H(b-x+v\Delta t)\lambda_{\mu }f_{\mu }(v)\;\text{d}v=\int_{(x-b)/\Delta t}^{\infty }\lambda_{\mu }f_{\mu }(v)\;\text{d}v\\ & =\frac{\lambda_{\mu }}{\sqrt{2\pi \sigma_{\mu }^{2}}}\int_{(x-b)/\Delta t}^{\infty }\exp\left(-\frac{v^{2}}{2\sigma_{\mu }^{2}}\right)\text{d}v=\frac{\lambda_{\mu }}{2}\;\text{erfc}\left(\frac{x-b}{\sigma_{\mu }\Delta t\sqrt{2}}\right). \end{array}$$

Thus, we proved (2.9). Integrating this formula over *x* in interval (b,∞), we obtain the average number of particles which are in the interval (b,∞) at time *t*=Δ*t*

$$\int_{b}^{\infty }\varrho (x;\Delta t,b)\;\text{d}x=\frac{\lambda_{\mu }}{2}\int_{0}^{\infty }\text{erfc}\left(\frac{z}{\sigma_{\mu }\Delta t\sqrt{2}}\right)\text{d}z=\frac{\lambda_{\mu }\sigma_{\mu }\Delta t}{\sqrt{2\pi }}.$$

Substituting (2.1) for λ_*μ*_ and (2.2) for *σ*_*μ*_, we obtain (2.7). □

We use lemmas 2.1 and 2.2 to design a computational test for multi-scale methods. As the number *n*≡*n*(*t*) of heat bath particles in [−*L*,*L*] is much larger than the number *N* of large particles, we will focus on models of a single large particle, i.e. *N*=1, which is described by its position *X* and velocity *V* . We choose a small time step Δ*t*. One iteration of the MD algorithm is presented in table 1. We first compute the positions of all particles at time *t*+Δ*t* in step [A1] by assuming that particles do not interact. Then we use (2.5) to incorporate collisions in step [A2]. As all heat bath particles have the same mass, the collisions between them result in exchange of colliding particles' positions and velocities. In particular, step [A2] can be implemented by sorting the heat bath particles during every iteration. If the large particle collides with a heat bath particle, then their positions at time *t*+Δ*t* will be obtained in step [A2] by calculating the exact time *t*_*c*_ of their collision, i.e. *t*_*c*_∈[*t*,*t*+Δ*t*]. Then we use their pre-collision velocities in the time interval [*t*,*t*_*c*_] and post-collision velocities in the time interval [*t*_*c*_,*t*+Δ*t*] to compute *X*(*t*+Δ*t*) and *x*^*i*^(*t*+Δ*t*). All heat bath particles which left the domain [−*L*,*L*] are removed in step [A3].

**Table 1.** One iteration of the computer implementation of MD model [A].

[A1]	Compute ‘free-flight positions’ of heat bath particles and the large particle at time *t*+Δ*t* by
	${\hat{x}}^{i}(t+\Delta t)=x^{i}(t)+v^{i}(t)\;\Delta t$ and $\hat{X}(t+\Delta t)=X(t)+V(t)\;\Delta t$.
[A2]	Compute post-collision velocities by (2.5) for every pair of particles which collided. Compute their post-collision positions *x*^*i*^(*t*+Δ*t*) and *X*(*t*+Δ*t*) by updating their ‘free-flight positions’ ${\hat{x}}^{i}(t+\Delta t)$ and $\hat{X}(t+\Delta t)$.
[A3]	Terminate trajectories of heat bath particles which left the domain [−*L*,*L*]. Update *n* accordingly.
[A4]	Generate a random number *r*_1_ uniformly distributed in (0,1).
	If *r*_1_<*γ*(*μ*+1)Δ*t*/8, then increase *n* by 1, and introduce a new heat bath particle at a position sampled according to the probability distribution proportional to *ϱ*(*x*;Δ*t*,−*L*). Its velocity is sampled according to the probability distribution proportional to *H*(−*L*−*x*+*vΔt*) *f*_*μ*_(*v*).
[A5]	Generate a random number *r*_2_ uniformly distributed in (0,1).
	If *r*_2_<*γ*(*μ*+1)Δ*t*/8, then increase *n* by 1, and introduce a new heat bath particle at position *x*^*n*^(*t*+Δ*t*) with velocity *v*^*n*^(*t*+Δ*t*) which are sampled according to probability distributions (2.11) and (2.12).
[A6]	Continue with step [A1] using time *t*=*t*+Δ*t*.

New heat bath particles are introduced in steps [A4] and [A5]. We assume that Δ*t* is chosen so small that (2.7) is much smaller than 1. Then (2.7) can be interpreted as a probability of introducing one particle from the left (resp. right) during one time step. Using lemma 2.2, the new particle will be introduced at the (left boundary) position which is sampled according to the probability distribution proportional to *ϱ*(*x*;Δ*t*,−*L*) in step [A4]. To sample from this probability distribution, we scale and shift a random number sampled from the complementary error function distribution *π* *erfc*(*z*), where z∈(0,∞). An acceptance–rejection algorithm for sampling random numbers from *π* *erfc*(*z*) is given in table 2. We use it with the constants *a*_1_ and *a*_2_ given by

2.10 $$a_{1}=0.532\;\text{and}\;a_{2}=0.814.$$

The values of constants *a*_1_ and *a*_2_ were numerically estimated to maximize the total acceptance probability $a_{2}/(a_{1}\sqrt{\pi })$ of the acceptance–rejection algorithm in table 2. Using (2.10), we accept 86% of proposed numbers *ζ*_2_.

**Table 2.** The acceptance–rejection algorithm which is used to sample random numbers which are distributed according to the probability distribution *π* *erfc*(*z*), where z∈(0,∞). In our simulations, we use constants *a*_1_ and *a*_2_ given by (2.10).

— Generate a random number *ζ*_1_ uniformly distributed in (0,1).
— Compute exponentially distributed random number *ζ*_2_ by $\zeta_{2}=-a_{1}\log(\zeta_{1})$.
— Generate a random number *ζ*_3_ uniformly distributed in (0,1).
— If *ζ*_1_*ζ*_3_<*a*_2_ *erfc*(*ζ*_2_), then choose *ζ*_2_ as a sample from the probability distribution *π* *erfc*(*z*). Otherwise, repeat the algorithm.

A particle introduced close to the right boundary in step [A5] will have its position sampled according to the probability distribution

2.11 $$C_{1}\;\mathrm{erfc}\left(\frac{L-x}{\sigma_{\mu }\Delta t\sqrt{2}}\right),\;\text{for}\;x\in (-\infty ,L),$$

where *C*_1_ is a normalization constant. The probability distribution (2.11) is proportional to *ϱ*(−*x*;Δ*t*,−*L*) and can be justified using the same argument as lemma 2.2. To sample from the probability distribution (2.11), we again use the acceptance–rejection algorithm in table 2 with parameters *a*_1_ and *a*_2_ given by (2.10). In step [A5], we also sample the velocity v∈R of the new particle using the truncated Gaussian distribution

2.12 $$C_{2}H(x-v\Delta t-L)\;f_{\mu }(v),$$

where *C*_2_ is a normalization constant. To sample random numbers according to the truncated normal distributions in steps [A4] and [A5], we use an acceptance–rejection algorithm which is derived as proposition 2.3 in [18].

**Figure 1.**
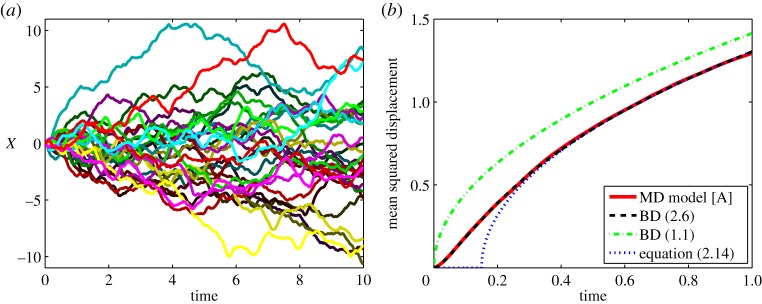
(*a*) Thirty illustrative trajectories of the heavy particle computed by the MD algorithm [A1]–[A6]. (*b*) The mean squared displacement computed by 10^3^ realizations of the algorithm [A1]–[A6] (solid line). The MD results are compared with BD results: equation (2.13) (dashed line), $\sqrt{2Dt}$ (dot-dashed line) and equation (2.14) (dotted line). We use *μ*=10^3^, *γ*=10, *D*=1, Δ*t*=10^−7^, *L*=20, *X*(0)=0 and *V* (0)=0. (Online version in colour.)

In figure 1, we present illustrative results computed by the algorithm [A1]–[A6]. We use *μ*=10^3^, *γ*=10 and *D*=1. We initialize the position and velocity of the heavy particle as *X*(0)=0 and *V* (0)=0 and we use the algorithm [A1]–[A6] with time step Δ*t*=10^−7^ in the interval [−*L*,*L*] where *L*=20. In figure 1*a*, we present 30 illustrative trajectories of the heavy particle *X*(*t*) computed for *t*∈[0,10]. The mean squared displacement given by the MD model [A] is plotted in figure 1*b* as the solid line. To illustrate the limiting result in lemma 2.1, we also plot the mean squared displacement corresponding to the limiting solution *X* of (2.6). It can be analytically computed as

2.13 $$\sqrt{E[{\left(X(t)-X(0)\right)}^{2}]}=\sqrt{2Dt-\frac{3D}{\gamma }+\frac{4D\exp[-\gamma t]}{\gamma }-\frac{D\exp[-2\gamma t]}{\gamma }},$$

where E[⋅] denotes the expected value. It is plotted as the dashed line in figure 1*b*. We also plot the mean squared displacement corresponding to the overdamped limit (1.1), i.e. $\sqrt{2Dt}$, as the dot-dashed line in figure 1*b*. If we neglect the exponential terms in (2.13), we obtain

2.14 $$\sqrt{E[{\left(X(t)-X(0)\right)}^{2}]}\approx \sqrt{2D\left(t-\frac{3}{2\gamma }\right)}.$$

This approximation is plotted in figure 1*b* as the dotted line. We will use (2.14) later in §6 to couple the overdamped BD model (1.1) with MD simulations.

## 3. Three-dimensional molecular dynamics models [B] and [C]

MD models [B] and [C] are three-dimensional generalizations of the MD model [A]. They are described in terms of positions **x**^*i*^ and velocities **v**^*i*^, *i*=1,2,3,…, of heat bath particles, and positions $X_{\mu }^{i}=[X_{\mu ;1}^{i},X_{\mu ;2}^{i},X_{\mu ;3}^{i}]$ and velocities $V_{\mu }^{i}=[V_{\mu ;1}^{i},V_{\mu ;2}^{i},V_{\mu ;3}^{i}]$, *i*=1,2,…,*N*, of heavy particles of mass *M*≫*m*, where *m* is the mass of a heat bath particle [16]. We again define *μ* by (1.5). We will denote by *R* the radius of a heavy particle.

MD models [B] and [C] are both based on elastic collisions of heavy molecules (balls with mass *M* and radius *R*) with point bath particles with masses *m*. As the collisions are without friction, conservation of momentum and energy then yields the following generalization of formulae (2.5) for post-collision velocities [16]

3.1 $$\begin{array}{rl} {\tilde{V}}_{\mu }^{i} & ={\left[V_{\mu }^{i}\right]}^{∥}+\frac{\mu -1}{\mu +1}{\left[V_{\mu }^{i}\right]}^{⊥}+\frac{2}{\mu +1}{\left[v^{j}\right]}^{⊥}, \end{array}$$

3.2 $$\begin{array}{rl} {\tilde{v}}^{j} & ={\left[v^{j}\right]}^{∥}+\frac{1-\mu }{\mu +1}{\left[v^{j}\right]}^{⊥}+\frac{2\mu }{\mu +1}{\left[V_{\mu }^{i}\right]}^{⊥}, \end{array}$$

where **v**^*j*^ is the velocity of the heat bath molecule which collided with the *i*th heavy molecule, tildes denote post-collision velocities, superscripts ⊥ denote projections of velocities on the line through the centre of the molecule and the collision point on its surface, and superscripts ∥ denote tangential components.

### (a) Molecular dynamics model [B]

MD model [B] will use the normal distribution for velocities of heat bath particles. The following lemma generalizes lemma 2.1 to the three-dimensional MD model [B].

**Lemma 3.1**

*Let* *γ*>0, *D*>0 *and* *R*>0. *Let us consider the MD model [B] where heat bath particles are distributed according to the Poisson distribution with density*

3.3 $$\lambda_{\mu }=\frac{3}{8R^{2}}\sqrt{\frac{(\mu +1)\gamma }{2\pi D}}.$$

*Let the velocities of heat bath particles be distributed according to*

3.4 $$f_{\mu }(v)=\frac{1}{\sigma_{\mu }^{3}{\left(2\pi \right)}^{3/2}}\exp\left[-\frac{v_{1}^{2}+v_{2}^{2}+v_{3}^{2}}{2\sigma_{\mu }^{2}}\right],\;\text{where}\;\sigma_{\mu }=\sqrt{(\mu +1)D\gamma }$$

*and* **v**=[*v*_1_,*v*_2_,*v*_3_]. *We will consider one heavy molecule in such a heat bath, i.e*. *N*=1. *Then the position and velocity of the heavy molecule*, **X**_*μ*_ *and* **V**_*μ*_, *converge (in the sense of distribution) to the solution of* *(1.6)* *and* *(1.7)* *in the limit* μ→∞.

**Proof.**

The MD model [B] and heat bath distributions (3.3) and (3.4) satisfy the assumptions of theorem 2.1 in Dürr *et al.* [16]. Their theorem expresses the limiting equation of a process with given λ_*μ*_ and *f*_*μ*_(**v**) in terms of moments of *f*_*μ*_. These moments can be analytically evaluated to verify the statement of lemma 3.1. We will also rederive this result in §5 as a consequence of the analysis of multi-scale methods. □

Lemma 3.1 can be viewed as a different formulation of theorem 2.1 in [16]. They were interested in the limit *m*→0, which is equivalent to μ→∞. Considering the scaling *m*^3/2^*f*(**v***m*^1/2^) of the velocity distribution of heat bath particles (with density scaled as λ/*m*^1/2^), they derived formulae for *γ* and *D* in terms of moments of *f* and λ. To formulate lemma 3.1, we inverted their results by deriving the appropriate distributions (3.3) and (3.4) which lead to the limiting BD model with a given *D* and *γ*.

### (b) Molecular dynamics model [C]

In lemma 3.1, we used the normal distribution for velocities (3.4). Another option is to use heat bath particles with fixed speed as is done in the following lemma 3.2. We denote the resulting MD model as the MD model [C].

**Lemma 3.2**

*Let* *γ*>0, *D*>0 *and* *R*>0. *Let us consider the MD model [C] where heat bath particles are distributed according to the Poisson distribution with density*

3.5 $$\lambda_{\mu }=\frac{3}{8\pi R^{2}}\sqrt{\frac{(\mu +1)\gamma }{D}}.$$

*Let the velocities of heat bath particles be distributed according to*

3.6 $$f_{\mu }(v)=\frac{1}{4\pi \sigma_{\mu }^{2}}\delta \left(\sqrt{v_{1}^{2}+v_{2}^{2}+v_{3}^{2}}-\sigma_{\mu }\right),\;\text{where}\;\sigma_{\mu }=2\sqrt{(\mu +1)D\gamma }$$

*and* *δ* *is the Dirac delta function. Let us consider one heavy molecule in this heat bath at position* **X**_*μ*_ *with velocity* **V**_*μ*_. *Then* **X**_*μ*_ *and* **V**_*μ*_ *converge (in the sense of distribution) to the solution of* *(1.6)* *and* *(1.7)* *in the limit* μ→∞.

Lemma 3.2 can again be proved using theorem 2.1 in [16] that is applicable to any spherically symmetric velocity distribution which has at least four finite moments.

### (c) Boundary conditions for molecular dynamics models [B] and [C]

Next, we generalize lemma 2.2 to the three-dimensional case. This will help us to specify boundary conditions for simulations which use the MD models [B] and [C] in finite domains.

**Lemma 3.3**

*Let* b∈R *and* Δ*t*>0. *Let us assume that heat bath particles are distributed according to the Poisson distribution with density* λ_*μ*_ *in the half space* $(-\infty ,b)\times R^{2};$ *their initial velocities are distributed according to* *f*_*μ*_(**v**) *and there are no particles in the half space* $(b,\infty )\times R^{2}$ *at time* *t*=0. *Let us assume that* λ_*μ*_ *and* *f*_*μ*_(**v**) *are either given by* *(3.3)* *and* *(3.4)* (*MD model* [*B*]) *or given by* *(3.5)* *and* *(3.6)* (*MD model* [*C*]).

*Then the positions* **x** *and velocities* **v** *of heat bath particles in the half space* $(b,\infty )\times R^{2}$ *are distributed at time* *t*=Δ*t* *according to*

3.7 $$H(b-x_{1}+v_{1}\Delta t)\;\lambda_{\mu }\;f_{\mu }(v),$$

*and the average number of particles in the semi-infinite cuboid* $(b,\infty )\times {\left(0,1\right)}^{2}$ *at time* *t*=Δ*t* is

3.8 $$\frac{3\gamma (\mu +1)\Delta t}{16\pi R^{2}}.$$

**Proof.**

Formula (3.7) is a generalization of formula (2.8) in lemma 2.2 and can be justified using the same arguments. To prove (3.8), we will distinguish two cases.

First, let us consider that λ_*μ*_ and *f*_*μ*_(**v**) are given by (3.3) and (3.4). Integrating (3.7) over positions and velocities (see the proof of lemma 2.2), we conclude that the average number of particles in the semi-infinite cuboid $(b,\infty )\times {\left(0,1\right)}^{2}$ at time *t*=Δ*t* is equal to

$$\frac{\lambda_{\mu }\sigma_{\mu }\Delta t}{\sqrt{2\pi }}=\frac{3\gamma (\mu +1)\Delta t}{16\pi R^{2}},$$

which is formula (3.8).

Next, let us consider that λ_*μ*_ and *f*_*μ*_(**v**) are given by (3.5) and (3.6). Integrating (3.7) with respect to **v**, we get the density of particles at $x\in (b,\infty )\times R^{2}$ at time *t*=Δ*t*,

3.9 $$\begin{array}{rl} \varrho (x;\Delta t,b) & =\int_{R^{3}}H(b-x_{1}+v_{1}\Delta t)\lambda_{\mu }f_{\mu }(v)\;\text{d}v\\ & =\frac{\lambda_{\mu }}{4\pi \sigma_{\mu }^{2}}\int_{(x_{1}-b)/\Delta t}^{\infty }\left(\int_{R^{2}}\delta \left(\sqrt{v_{1}^{2}+v_{2}^{2}+v_{3}^{2}}-\sigma_{\mu }\right)\;\text{d}v_{2}\;\text{d}v_{3}\;\right)\text{d}v_{1}\\ & =\frac{\lambda_{\mu }}{2\sigma_{\mu }}{\left(\sigma_{\mu }-\frac{x_{1}-b}{\Delta t}\right)}_{+}, \end{array}$$

where (⋅)_+_ denotes a positive part. Integrating this formula over **x** in the semi-infinite cuboid $(b,\infty )\times {\left(0,1\right)}^{2}$, we obtain

$$\begin{array}{rl} \int_{(b,\infty )\times {\left(0,1\right)}^{2}}\varrho (x;\Delta t,b)\;\text{d}x & =\frac{\lambda_{\mu }}{2\sigma_{\mu }}\int_{b}^{\infty }{\left(\sigma_{\mu }-\frac{x_{1}-b}{\Delta t}\right)}_{+}\;\text{d}x_{1}\\ & =\frac{\lambda_{\mu }}{2\sigma_{\mu }}\int_{0}^{\sigma_{\mu }\Delta t}\left(\sigma_{\mu }-\frac{x_{1}}{\Delta t}\right)\text{d}x_{1}=\frac{\lambda_{\mu }\sigma_{\mu }\Delta t}{4}. \end{array}$$

Substituting (3.5) for λ_*μ*_ and (3.6) for *σ*_*μ*_, we obtain (3.8). □

**Figure 2.**
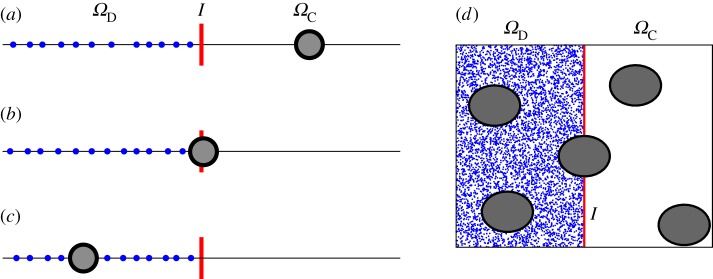
(*a*–*c*) Schematic of one-dimensional multi-scale set-up (1.4). (*d*) Schematic of multi-scale set-up (1.4) (in two dimensions). (Online version in colour.)

Lemma 3.3 can be used to specify boundary conditions for simulations of the MD models [B] and [C] in finite domains as we did for the one-dimensional case in lemma 2.2. In §5, we will use lemma 3.3 to develop and analyse multi-scale approaches which can efficiently and accurately compute results with an MD level of detail in a (relatively small) subdomain *Ω*_D_⊂*Ω* by using coarser BD simulations in the remainder. The geometry of the desired multi-scale method is formulated using (1.4) where an MD model is used in *Ω*_D_, a coarser BD model is used in *Ω*_C_ and these models are coupled across the interface *I*. The situation is schematically shown in figure 2*d*, which presents a two-dimensional version of our multi-scale set-up. Here, point particles describe heat bath molecules which are used in *Ω*_D_. Large biomolecules of interest are denoted as grey circles. They are simulated using BD in *Ω*_C_. The solid line denotes interface *I*.

The schematic in figure 2*d* is presented in two spatial dimensions to better visualize the problem geometry. MD models [B] and [C] are formulated in a three-dimensional physical space. In the three-dimensional version of figure 2*d*, the cloud of point particles would cover the grey ball. To get some insights into this multi-scale problem, we start with the one-dimensional MD model [A].

## 4. From one-dimensional molecular dynamics model [A] to Brownian dynamics

In the case of one-dimensional MD model [A], the situation is schematically shown in figure 2*a*–*c*, where we consider only one large (heavy) particle, i.e. *N*=1. The large particle can either be in *Ω*_C_ (figure 2*a*), or be in *Ω*_D_ (figure 2*c*) or crossing the boundary as is shown in figure 2*b*. Our geometry is given by (1.4), where

$$\Omega =(-L,L),\;\Omega_{D}=(-L,0),\;\Omega_{C}=(0,L)\;\text{and}\;I=\{0\}.$$

The large particle covers the interval (*X*(*t*)−*R*,*X*(*t*)+*R*). Let us consider that the large particle intersects the interface *I*, as is shown in figure 2*b*. Then *I*⊂(*X*(*t*)−*R*,*X*(*t*)+*R*), which is equivalent to *X*(*t*)∈(−*R*,*R*). The heat bath particles are simulated in *Ω*_D_ using the MD model [A]. Let us choose Δ*t* so small that the probability of two collisions happening in the time interval (*t*,*t*+Δ*t*) is negligible. As we do not explicitly simulate heat bath particles in *Ω*_C_, we will consider an additional correction of the velocity of the heavy particle in the form

4.1 $$V(t+\Delta t)=\tilde{V}(t+\Delta t)+\alpha (V(t))\Delta t+\beta (V(t))\sqrt{\Delta t}\;\xi ,$$

where $\tilde{V}(t+\Delta t)$ is the post-collision velocity of the heavy particle at time *t*+Δ*t*, which only takes into account collisions with the heat bath particles from the left. It is either equal to *V* (*t*) or computed by (2.5) if a collision with a heat bath particle occurred in *Ω*_D_. Equation (4.1) is adding both the drift term *α*(*V* (*t*))Δ*t* and the noise term $\beta (V(t))\sqrt{\Delta t}\;\xi$, where ξ is a normally distributed random number with zero mean and unit variance. The drift and noise terms implicitly take into account collisions at the right boundary (*X*(*t*)+*R*) of the heavy particle. Passing Δ*t*→0, we observe that the contributions of the collisions at the right boundary are given by the Itō stochastic differential equation

4.2 dV=α(V) dt+β(V) dW.

If we explicitly modelled heat bath particles in *Ω*_C_, then they would be distributed according to the Poisson distribution with density λ_*μ*_ in the interval (X(t)+R,∞). Their initial velocities would be given according to (2.2). Thus, using lemma 2.2 and (2.5), we can estimate the drift coefficent of the stochastic differential equation (4.2) to get

$$\alpha (V)=\frac{1}{\Delta t}\int_{X(t)+R}^{\infty }\int_{-\infty }^{\infty }\frac{2(v-V)}{\mu +1}\;H\left(\frac{X(t)+R-x}{\Delta t}+V-v\right)\lambda_{\mu }f_{\mu }(v)\;\text{d}v\;\text{d}x,$$

where *H*(⋅) is the Heaviside step function. Using (2.2), we obtain

4.3 $$\begin{array}{rl} \alpha (V) & =\frac{2\lambda_{\mu }}{\Delta t(\mu +1)\sigma_{\mu }\sqrt{2\pi }}\int_{0}^{\infty }\int_{-\infty }^{V-x/\Delta t}(v-V)\exp\left(-\frac{v^{2}}{2\sigma_{\mu }^{2}}\right)\text{d}v\;\text{d}x\\ & =-\frac{\lambda_{\mu }}{\mu +1}\left((\sigma_{\mu }^{2}+V^{2})\;\text{erfc}\left[-\frac{V}{\sigma_{\mu }\sqrt{2}}\right]+\frac{V\sigma_{\mu }\sqrt{2}}{\sqrt{\pi }}\exp\left[-\frac{V^{2}}{2\sigma_{\mu }^{2}}\right]\right), \end{array}$$

where λ_*μ*_ and *σ*_*μ*_ are given by (2.1) and (2.2). In the limit μ→∞, we have $V/\sqrt{\mu +1}\to 0$. Thus, we use the Taylor expansion in (4.3) to get

4.4 $$\alpha (V)\approx -\frac{\gamma \sqrt{\pi (\mu +1)D\gamma }}{4\sqrt{2}}-\frac{\gamma }{2}V-\frac{\sqrt{\pi \gamma }}{4\sqrt{2D(\mu +1)}}V^{2}.$$

The noise term in (4.2) can be computed by

$$\beta^{2}(V)=\frac{1}{\Delta t}\int_{X(t)+R}^{\infty }\int_{-\infty }^{\infty }\frac{4{\left(v-V\right)}^{2}}{{\left(\mu +1\right)}^{2}}\;H\left(\frac{X(t)+R-x}{\Delta t}+V-v\right)\lambda_{\mu }f_{\mu }(v)\;\text{d}v\;\text{d}x.$$

Using (2.2), we obtain

$$\begin{array}{rl} \beta^{2}(V) & =\frac{4\lambda_{\mu }}{\Delta t{\left(\mu +1\right)}^{2}\sigma_{\mu }\sqrt{2\pi }}\int_{0}^{\infty }\int_{-\infty }^{V-x/\Delta t}{\left(v-V\right)}^{2}\exp\left(-\frac{v^{2}}{2\sigma_{\mu }^{2}}\right)\text{d}v\;\text{d}x\\ & =\frac{2\lambda_{\mu }}{{\left(\mu +1\right)}^{2}}\left(V(3\sigma_{\mu }^{2}+V^{2})\;\text{erfc}\left[-\frac{V}{\sigma_{\mu }\sqrt{2}}\right]+\frac{2(2\sigma_{\mu }^{2}+V^{2})\sigma_{\mu }}{\sqrt{2\pi }}\exp\left[-\frac{V^{2}}{2\sigma_{\mu }^{2}}\right]\right). \end{array}$$

Using (2.1), (2.2) and the Taylor expansion, we obtain

4.5 $$\beta (V)\approx \sqrt{\gamma^{2}D+\frac{3\gamma \sqrt{\pi D\gamma }}{2\sqrt{2(\mu +1)}}V+\frac{3\gamma }{2(\mu +1)}V^{2}}.$$

Equations (4.4) and (4.5) are used in the multi-scale algorithm in table 3. The first two steps [M1] and [M2] are the same as [A1] and [A2]. As heat bath particles are only simulated in the subdomain *Ω*_D_=(−*L*,0), we remove all particles which left *Ω*_D_ during the time interval (*t*,*t*+Δ*t*) in step [M3]. Step [M4] is the same as [A4], which introduces heat bath particles that have entered *Ω*_D_ through its left boundary *x*=−*L* during the time interval (*t*,*t*+Δ*t*). The boundary at *x*=0 is treated in step [M5] if the heavy particle does not intersect with this boundary. We assume that Δ*t* is chosen so small that (2.7) is much smaller than 1. Then (2.7) can be interpreted as a probability of introducing one particle from the left (resp. right) during one time step. A particle introduced close to the right boundary of *Ω*_D_ in step [M5] will have its position sampled according to the probability distribution

4.6 $$C_{1}\;\mathrm{erfc}\left(\frac{-x}{\sigma_{\mu }\Delta t\sqrt{2}}\right),\;\text{for}\;x\in (-\infty ,0),$$

where *C*_1_ is a normalization constant. The probability distribution (4.6) can be justified using the same argument as lemma 2.2 and equation (2.11). To sample from the probability distribution (4.6), we again use the acceptance–rejection algorithm in table 2 with parameters *a*_1_ and *a*_2_ given by (2.10). In step [M5], we also sample the velocity v∈R of the new particle using the truncated Gaussian distribution

4.7 $$C_{2}H(x-v\Delta t)\;f_{\mu }(v),$$

where *C*_2_ is a normalization constant. To sample random numbers according to the truncated normal distributions in steps [M4] and [M5], we again use the acceptance–rejection algorithm which is derived as proposition 2.3 in [18]. If the heavy particle does intersect with the boundary *I*, then step [M6] is executed. It uses (4.1) to incorporate collisions of heat bath particles from the right. If the particle does not intersect with *Ω*_D_, then we simulate it in step [M7] using the discretized version of (1.7) given by

4.8 $$V(t+\Delta t)=-\gamma V(t)\Delta t+\gamma \sqrt{2D\Delta t}\;\xi .$$

**Table 3.** One iteration of the computer implementation of the multi-scale algorithm which is based on the MD model [A].

[M1]	Compute ‘free-flight positions’ of heat bath particles and the heavy particle at time *t*+Δ*t* using step [A1].
[M2]	Compute post-collision velocities by (2.5) for every pair of particles which collided using step [A2].
[M3]	Terminate trajectories of heat bath particles which left the subdomain *Ω*_D_=(−*L*,0). Update *n* accordingly.
[M4]	Implement the influx of heat bath particles through the boundary *x*=−*L* using step [A4].
[M5]	If *X*(*t*)∉(−*R*,*R*), then generate a random number *r*_2_ uniformly distributed in (0,1). If *r*_2_<*γ*(*μ*+1)Δ*t*/8, then increase *n* by 1, and introduce a new heat bath particle at position *x*^*n*^(*t*+Δ*t*) with velocity *v*^*n*^(*t*+Δ*t*) which are sampled according to probability distributions (4.6) and (4.7).
[M6]	If *X*(*t*)∈(−*R*,*R*), then update the heavy particle velocity using (4.1).
[M7]	If *X*(*t*)∈[*R*,*L*), then update the velocity of the heavy particle using (4.8).
[M8]	Continue with step [M1] using time *t*=*t*+Δ*t*.

**Figure 3.**
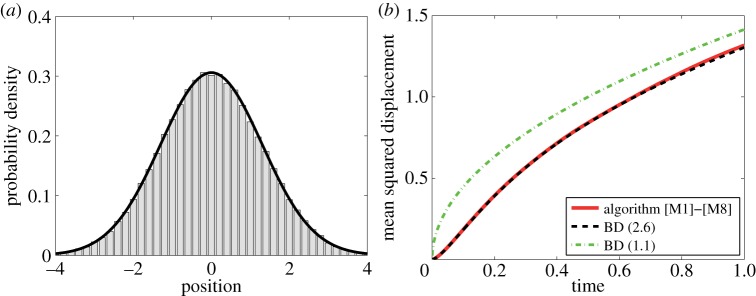
(*a*) Probability distribution of the heavy particle at time *t*=1 computed by the multi-scale algorithm [M1]–[M8] (grey histogram) is compared with the distribution (4.9) given by the BD model (2.6) (black solid line). (*b*) The time evolution of the mean squared displacement computed by 10^5^ realizations of the algorithm [M1]–[M8] (solid line) is compared with equation (2.13) (dashed line) and $\sqrt{2Dt}$ (dot-dashed line). We use *μ*=10^3^, *γ*=10, *D*=1, Δ*t*=10^−7^, *L*=10, *R*=1, *X*(0)=0 and *V* (0)=0. (Online version in colour.)

In figure 3, we present illustrative results computed by the algorithm [M1]–[M8]. We consider one heavy particle which starts at position *X*(0)=0 with velocity *V* (0)=0, as we did in figure 1. The distribution of its position at time *t*=1, computed using 10^5^ realizations of the algorithm [M1]–[M8], is plotted in figure 3*a*. It is compared with the distribution obtained by the limiting BD model (2.6), which is, for *t*≫*γ*^−1^, given by [19]

4.9 $$\frac{1}{\sqrt{4\pi D(t-t^{*})}}\exp\left[-\frac{x^{2}}{4D(t-t^{*})}\right],\;\text{where}\;t^{*}=\frac{3}{2\gamma }.$$

In figure 3*b*, we plot the time evolution of the mean squared displacement. This figure can be directly compared with figure 1*b*, because we use the same parameter values. The results computed by the multi-scale algorithm [M1]–[M8] compare well with the results given by the BD model (2.6). We have already shown in figure 1*b* that the limiting BD model (2.6) also compares well with the MD simulations. In particular, the algorithm [M1]–[M8] is able to compute results with the MD-level precision by using coarser BD models in a part of the computational domain.

The stochastic differential equation (4.2) was derived for collisions from the right. Using the same argument, we can also derive a stochastic differential equation which is approximating the effect of collisions from the left. We obtain

4.10 dV=−α(−V) dt+β(−V) dW.

Adding (4.2) and (4.10) and using the independence of noise terms in (4.2) and (4.10), we can approximate collisions from both sides by the following stochastic differential equation for the velocity of the heavy particle:

4.11 $$\text{d}V=(\alpha (V)-\alpha (-V))\;\text{d}t+\sqrt{\beta^{2}(V)+\beta^{2}(-V)}\;\text{d}W.$$

Substituting (4.4) and (4.5), we derive (1.7). In particular, we have verified the limiting result in lemma 2.1.

## 5. From three-dimensional molecular dynamics models [B] and [C] to Brownian dynamics

We use a simple multi-scale geometry where domain $\Omega =R^{3}$ is divided into two half spaces. Heavy molecules are simulated in both half spaces. In $\Omega_{D}=(-\infty ,0)\times R^{2}$, we use the MD model [B] or [C]. It is coupled with the BD model given by (1.6) and (1.7) in $\Omega_{C}=(0,\infty )\times R^{2}.$ This set-up is a three-dimensional version of multi-scale problems which are schematically drawn in figure 2. Boundary conditions for heat bath particles at the interface $I=\{0\}\times R^{2}$ can be specified using lemma 3.3.

As in §4, we need to analyse the behaviour of a heavy molecule when it intersects with the interface *I*. Such a molecule is subject to the collisions with heat bath particles on the part of its surface which lies in *Ω*_D_. This has to be compensated by using a suitable random force from *Ω*_C_, so that the overall model is equivalent to (1.6) and (1.7) in the BD limit. To simplify the presentation of the algorithm, we use the same time step in *Ω*_D_ and *Ω*_C_. In §6, we present coupling of three-dimensional MD models with the BD model (1.1), which will make use of different time steps in different parts of the computational domain.

The heavy particle is the ball with centre **X**=[*X*_1_,*X*_2_,*X*_3_] with velocity **V**=[*V*_1_,*V*_2_,*V*_3_] and radius *R*. It intersects the interface *I* if *X*_1_(*t*)∈(−*R*,*R*). Let us consider that heat bath particles are simulated in *Ω*_D_ using the MD model [B] or the MD model [C]. Let us choose Δ*t* so small that the probability of two collisions happening in the time interval (*t*,*t*+Δ*t*) is negligible. As we do not explicitly simulate the heat bath particles in *Ω*_C_, we will consider an additional correction of the velocity of the heavy particle in the form

5.1 $$V(t+\Delta t)=\tilde{V}(t+\Delta t)+\alpha (X(t),V(t))\Delta t+\beta (X(t),V(t))\sqrt{\Delta t}\;\xi ,$$

where $\tilde{V}(t+\Delta t)$ is the post-collision velocity of the heavy particle at time *t*+Δ*t*, which only takes into account collisions with the heat bath particles from *Ω*_D_. It is either equal to **V**(*t*) or computed by (3.1) if a collision with a heat bath particle occurred in *Ω*_D_. Note that we dropped the subscript *μ* in (3.1) and (3.2) to simplify our notation. Equation (5.1) is a generalization of (4.1) to three-dimensional simulations where ***α***(**X**(*t*),**V**(*t*))Δ*t* is the drift vector, $\beta (X(t),V(t))\sqrt{\Delta t}\;\xi$ is the noise term and **ξ**=[ξ_1_,ξ_2_,ξ_3_] is the vector of three normally distributed random numbers with zero mean and unit variances. Passing Δ*t*→0, we observe that the contributions of the collisions from *Ω*_D_ are given by the Itō stochastic differential equation

5.2 dV=α(X(t),V(t)) dt+β(X(t),V(t)) dW.

To estimate drift ***α*** and diffusion coefficient ***β***, we separately consider MD models [B] and [C] in §5*a* and §5*b*, respectively.

### (a) Molecular dynamics model [B]

The following lemma will be useful to estimate the drift coefficient ***α***.

**Lemma 5.1**

*Let* *γ*>0, *D*>0, *R*>0 *and* Δ*t*>0. *Let us consider the MD model [B] where the positions and velocities of heat bath particles are distributed according to* (3.3) *and* (3.4). *Let us consider one heavy molecule in such a heat bath, i.e.* *N*=1, *with the position of its centre to be at* **X**(*t*)=[*X*_1_(*t*),*X*_2_(*t*),*X*_3_(*t*)] *and with velocity* **V**(*t*)=[*V*_1_(*t*),*V*_2_(*t*),*V*_3_(*t*)]. *Let* **y**=(*y*_1_,*y*_2_,*y*_3_) *be a given point on the surface of the heavy molecule at time* *t*, *i.e.*

5.3 $${\left(y_{1}-X_{1}(t)\right)}^{2}+{\left(y_{2}-X_{2}(t)\right)}^{2}+{\left(y_{3}-X_{3}(t)\right)}^{2}=R^{2}.$$

*Then the average change of the* *j*-th *component of the velocity of the heavy molecule caused by collisions with heat bath particles in the time interval* (*t*,*t*+Δ*t*) *at the surface area* (**y**,**y**+*d***y**) is *ψ*_*j*_(**y**) *d***y**, where

5.4 $$\begin{array}{rl} \psi_{j}(y) & =-\frac{\lambda_{\mu }\sigma_{\mu }^{2}(y_{j}-X_{j}(t))\Delta t}{(\mu +1)R}+\frac{4\lambda_{\mu }\sigma_{\mu }(y_{j}-X_{j}(t))\Delta t}{(\mu +1)R^{2}\sqrt{2\pi }}V(t)\cdot (y-X(t))+O(∥V{∥}^{2}). \end{array}$$

**Proof.**

Let us consider that a heat bath particle which was at point **x** at time *t* collided with the heavy molecule at time *t*+*τ*∈(*t*,*t*+Δ*t*) at the surface point which had coordinate **y** at time *t*. Then the coordinate of the surface point at the collision time *t*+*τ* was **y**+*τ***V**(*t*) and the pre-collision velocity of the heat bath molecule was **v**=**V**(*t*)+(**y**−**x**)/*τ*. Using equation (3.1), we can write the change of the velocity of the heavy molecule during the collision as

5.5 $$\frac{2}{\mu +1}{\left[v-V(t)\right]}^{⊥}=\frac{2}{\mu +1}\left(\frac{\left(y-x\right)}{\tau }\cdot \frac{\left(y-X(t)\right)}{R}\right)\frac{\left(y-X(t)\right)}{R}.$$

The position **x** of the heat bath particle must be in the half space which lies above the plane tangent to the heavy molecule at the collision point **y**+*τ***V**(*t*). It can be parametrized by

$$x=y+\tau \;V(t)+c_{1}\tau \frac{\left(y-X(t)\right)}{R}+c_{2}\tau \eta_{2}+c_{3}\tau \eta_{3},$$

where *c*_1_>0, $c_{2}\in R,$ $c_{3}\in R,$ and (**y**−**X**(*t*))/*R*, ***η*_2_**, ***η*_3_** is the orthornormal basis in $R^{3}.$ Then (5.5) reads as follows:

$$\frac{2}{\mu +1}{\left[v-V(t)\right]}^{⊥}=-\frac{2}{(\mu +1)R}\left(c_{1}+V(t)\cdot \frac{\left(y-X(t)\right)}{R}\right)(y-X(t)).$$

Thus, we have

5.6 $$\begin{array}{rl} \psi_{j}(y) & =-\frac{2\lambda_{\mu }(y_{j}-X_{j}(t))}{(\mu +1)R}\int_{0}^{\infty }\int_{-\infty }^{\infty }\int_{-\infty }^{\infty }\int_{0}^{\Delta t}{\left(c_{1}+V(t)\cdot \frac{\left(y-X(t)\right)}{R}\right)}^{2}\\ & \;\times f_{\mu }\left(-c_{1}\frac{y-X(t)}{R}-c_{2}\eta_{2}-c_{3}\;\eta_{3}\right)\text{d}\tau \;\text{d}c_{3}\;\text{d}c_{2}\;\text{d}c_{1}. \end{array}$$

Substituting (3.4) for *f*_*μ*_ and integrating over *τ*, *c*_2_ and *c*_3_, we have

$$\psi_{j}(y)=-\frac{\lambda_{\mu }(y_{j}-X_{j}(t))\Delta t\sqrt{2}}{(\mu +1)R\sigma_{\mu }\sqrt{\pi }}\int_{0}^{\infty }{\left(c_{1}+V(t)\cdot \frac{\left(y-X(t)\right)}{R}\right)}^{2}\exp\left[-\frac{c_{1}^{2}}{2\sigma_{\mu }^{2}}\right]\;\text{d}c_{1}.$$

Integrating over *c*_1_, we deduce (5.4). □

Using lemma 5.1, we can compute the drift coefficient ***α***(**X**(*t*),**V**(*t*)) in equation (5.2) as follows:

5.7 $$\alpha_{j}(X(t),V(t))=\frac{1}{\Delta t}\int_{S(X(t))}\psi_{j}(y)\;\text{d}y,$$

where *S*(**X**(*t*)) is the part of the surface of the heavy molecule which intersects the BD subdomain *Ω*_C_, i.e.

$$S(X(t))=\{y\in \Omega_{C}|y\;\text{satisfies}\;(\text{5.3})\}.$$

Substituting (5.4) into (5.7), we have

$$\begin{array}{r} \alpha_{j}(X(t),V(t))=-\frac{\lambda_{\mu }\sigma_{\mu }^{2}}{(\mu +1)R}\int_{S(X(t))}(y_{j}-X_{j}(t))\;\text{d}y-\frac{4\lambda_{\mu }\sigma_{\mu }V_{j}(t)}{(\mu +1)R^{2}\sqrt{2\pi }}\int_{S(X(t))}(y_{j}-X_{j}{\left(t)\right)}^{2}\;\text{d}y. \end{array}$$

Using (3.3) and (3.4) and evaluating the surface integrals, we obtain

5.8 $$\alpha_{1}(X,V)=-\frac{3\gamma \sqrt{\pi (\mu +1)D\gamma }}{8\sqrt{2}}\left(1-\frac{X_{1}^{2}}{R^{2}}\right)-\frac{\gamma V_{j}}{2}\left(1+\frac{X_{1}^{3}}{R^{3}}\right)$$

and

5.9 $$\alpha_{j}(X,V)=-\frac{\gamma V_{j}}{4}\left(2+3\frac{X_{1}}{R}-\frac{X_{1}^{3}}{R^{3}}\right),\;\text{for}\;j=2,3,$$

where we dropped the dependence on time *t* to shorten the resulting formulae. The noise matrix ***β***(**X**(*t*),**V**(*t*)) will be estimated using ***β***(**X**(*t*),**0**), i.e. we will only use the first term in the Taylor expansion in **V**. Using similar arguments as in the proof of (5.6) and (5.7), we have

5.10 $$\begin{array}{rl} \beta_{i,i}^{2}(X(t),0) & =-\frac{4\lambda_{\mu }}{{\left(\mu +1\right)}^{2}R^{2}}\int_{S(X(t))}\int_{0}^{\infty }\int_{-\infty }^{\infty }\int_{-\infty }^{\infty }c_{1}^{3}\;(y_{i}-X_{i}{\left(t)\right)}^{2}\\ & \;\times f_{\mu }\left(-c_{1}\frac{y-X(t)}{R}-c_{2}\eta_{2}-c_{3}\eta_{3}\right)\text{d}c_{3}\;\text{d}c_{2}\;\text{d}c_{1}\;\text{d}y, \end{array}$$

for *i*=1,2,3. Substituting (3.4) for *f*_*μ*_, (3.3) for λ_*μ*_ and using ***β***(**X**,**V**)=***β***(**X**,**0**), we obtain

5.11 $$\begin{array}{rl} & \beta_{1,1}(X,V)=\gamma \sqrt{D}\sqrt{1+\frac{X_{1}^{3}}{R^{3}}},\\ & \beta_{j,j}(X,V)=\gamma \sqrt{D}\sqrt{1+\frac{3X_{1}}{2R}-\frac{X_{1}^{3}}{2R^{3}}},\;\text{for}\;j=2,3\\ \;\text{and}\; & \beta_{i,j}(X,V)=0,\;\text{for}\;i\neq j, \end{array}\}$$

where the last equation can be verified using the same argument as equation (5.10). Note that, by substituting *X*_1_=*R* into (5.8), (5.9) and (5.11), we verify the limiting result in lemma 3.1.

### (b) Molecular dynamics model [C]

Equations (5.6), (5.7) and (5.10), which are derived in §5*a*, are applicable to both MD models [B] and [C]. To estimate the drift coefficient ***α***(**X**,**V**) for the MD model [C], we substitute (3.5) for λ_*μ*_ and (3.6) for *f*_*μ*_ in (5.6) and (5.7). We obtain

5.12 $$\alpha_{1}(X,V)=-\frac{\gamma \sqrt{(\mu +1)D\gamma }}{2}\left(1-\frac{X_{1}^{2}}{R^{2}}\right)-\frac{\gamma V_{j}}{2}\left(1+\frac{X_{1}^{3}}{R^{3}}\right),$$

and *α*_2_(**X**,**V**) and *α*_3_(**X**,**V**) are again given by (5.9). Substituting (3.5) and (3.6) in (5.10) and integrating, we obtain that noise matrix ***β***(**X**,**V**) satisfies (5.11). We again note that the special choice *X*_1_=*R* in (5.12) can be used to verify the limiting result in lemma 3.2.

### (c) Illustrative numerical results

In §5*a*,*b*, we have observed that the only difference between MD models [B] and [C] is a different formula for the coefficient *α*_1_(**X**,**V**) in (5.2), given by (5.8) and (5.12), respectively. The remaining terms in (5.2) are the same, given by (5.9) and (5.11). In this section, we present an illustrative computation with the MD model [C], but the same results can also be obtained with the MD model [B] (results not shown). An illustrative computation with the MD model [B] is presented later in §6.

**Figure 4.**
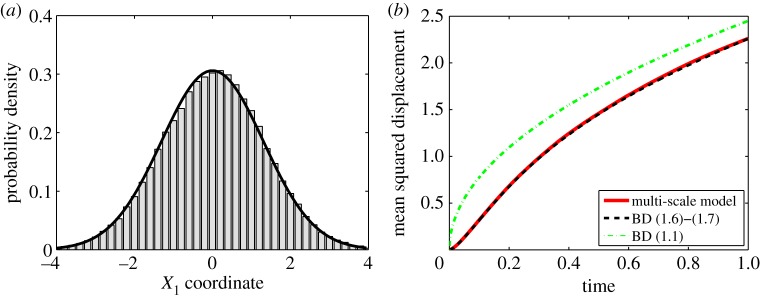
(*a*) Probability distribution of the first coordinate, *X*_1_, of the heavy particle at time *t*=1 computed by the three-dimensional multi-scale algorithm (grey histogram) is compared with the distribution (4.9) given by the BD model (black solid line). (*b*) The time evolution of the mean squared displacement computed by 10^5^ realizations of the three-dimensional multi-scale algorithm (solid line) is compared with the limiting BD model (1.6) and (1.7) (dashed line) and $\sqrt{6Dt}$ (dot-dashed line). We use *μ*=10^3^, *γ*=10, *D*=1, Δ*t*=10^−6^, *L*=5, *R*=1, **X**(0)=[0,0,0] and **V**(0)=[0,0,0]. (Online version in colour.)

We consider a three-dimensional generalization of the illustrative problem from figure 3. One heavy particle which starts at position **X**(0)=[0,0,0] with velocity **V**(0)=[0,0,0] is simulated using a three-dimensional generalization of the algorithm [M1]–[M8]. We use the MD model [C] in $\Omega_{D}=(-\infty ,0)\times R^{2}$ and the BD model (1.6) and (1.7) in $\Omega_{C}=(0,\infty )\times R^{2}.$ In step [M6], we replace (4.1) with its three-dimensional analogue (5.1), where drift ***α*** and diffusion coefficient ***β*** are given by (5.12), (5.9) and (5.11). The distribution of *X*_1_ positions of the heavy particle at time *t*=1, computed using 10^5^ realizations of the multi-scale algorithm, is plotted in figure 4*a*. The limiting BD result is again given by (4.9). In figure 4*b*, we plot the time evolution of the mean squared displacement. The mean squared displacement corresponding to the limiting BD model (1.6) and (1.7) is given by (2.13) multiplied by $\sqrt{3}\;$ because we have three spatial dimensions (dashed line). As expected, the models compare well. The mean squared displacement obtained for the BD model (1.1) is plotted as the dot-dashed line.

## 6. Application to protein binding to receptors

**Figure 5.**
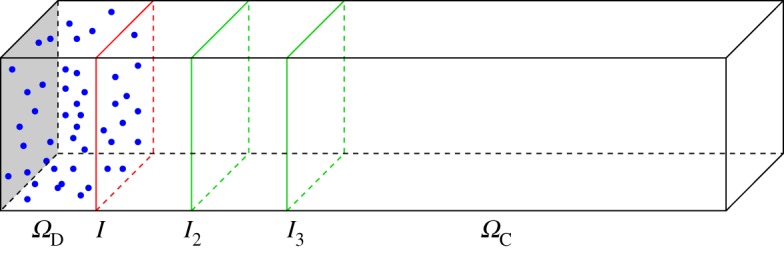
Schematic of the computational domain used in the protein binding example. (Online version in colour.)

In this section, we apply our results to a simplified model of protein binding to receptors on the cell membrane. We consider simple geometry, which is schematically shown in figure 5. Our computational domain is a part of the intracellular space next to the cell membrane given as the cuboid *Ω*=[0,*L*_1_]×[0,*L*_2_]×[0,*L*_2_], where *L*_1_>0 and *L*_2_>0. The cell membrane is modelled by one side of the cuboid, namely

$$\partial \Omega_{M}=\{0\}\times [0,L_{2}]\times [0,L_{2}],$$

which is shaded grey in figure 5. Our goal is to model the binding of diffusing proteins to receptors on the cell membrane with an MD level of detail. Therefore, we define *Ω*_D_ as a part of the intracellular space which is close to the cell membrane ∂*Ω*_M_, i.e.

$$\Omega_{D}=[0,h]\times [0,L_{2}]\times [0,L_{2}]\;\text{and}\;\Omega_{C}=[h,L_{1}]\times [0,L_{2}]\times [0,L_{2}],$$

where *h*>0 and the interface *I* is at *x*_1_=*h*. Diffusing proteins are modelled as spheres of radius *R*. We consider that a protein which hits the boundary ∂*Ω*_M_ will bind to a receptor with probability *P*, and otherwise it is reflected. This type of a reactive boundary condition is common for BD simulations [20]. In the case of MD, more detailed models of protein binding could be introduced in *Ω*_D_ [21,22]. However, the main goal of this section is to show how an MD model in *Ω*_D_ can be coupled with BD simulators which have been developed for simulations of intracellular processes. Therefore, we keep the MD model in *Ω*_D_ as simple as possible.

If we used BD model (1.6)–(1.7) in *Ω*_C_, then the situation would be more or less the same as in §5. However, modern BD simulators of intracellular processes work with the high-friction limit (1.1) rather than (1.6)–(1.7). For example, the software package Smoldyn discretizes (1.1) with a fixed time step and uses (1.2) to update positions of diffusing proteins. In particular, it uses larger values of time step than we used in §5. Then the problem can be formulated as follows: we would like to use the MD model with time step Δ*t* in *Ω*_D_ and couple it with the BD model (1.2) with larger time step $\bar{\Delta t}$, namely

6.1 $$X_{i}(t+\bar{\Delta t})=X_{i}(t)+\sqrt{2D\bar{\Delta t}}\;\xi_{i},\;i=1,2,3,$$

if the diffusing molecule is far away from *Ω*_D_. We couple these models using the intermediate BD model (1.6)–(1.7). We introduce two additional interfaces

$$I_{2}=\{h_{2}\}\times [0,L_{2}]\times [0,L_{2}]\;\text{and}\;I_{3}=\{h_{3}\}\times [0,L_{2}]\times [0,L_{2}],$$

where *h*<*h*_2_<*h*_3_<*L*_1_, as shown in figure 5. We denote

$$\Omega_{C1}=[h,h_{3}]\times [0,L_{2}]\times [0,L_{2}]\;\text{and}\;\Omega_{C2}=[h_{2},L_{1}]\times [0,L_{2}]\times [0,L_{2}],$$

i.e. *Ω*_*C*1_ and *Ω*_*C*2_ are two overlapping subdomains of *Ω*_C_. We simulate the time evolution of the position **X**(*t*) of one protein molecule. If **X**(*t*)∈*Ω*_*C*2_, then the BD model (6.1) will be used in *Ω*_*C*2_ until the molecule leaves *Ω*_*C*2_. Then we switch to the shorter time step Δ*t* and use the BD model (1.6)–(1.7) in *Ω*_*C*1_. The protein molecule can leave *Ω*_*C*1_ in two possible ways:

(i) The protein molecule crosses the interface *I*_3_.
  Then we revert to the BD model (6.1) which is used in *Ω*_*C*2_.
(ii) The protein molecule crosses the interface *I*.
  Then we use the method from §5 for coupling the MD model in *Ω*_D_ with the BD model (1.6)–(1.7) in *Ω*_*C*1_.

As the subdomains *Ω*_*C*1_ and *Ω*_*C*2_ overlap, we can use the limiting result (4.9), which implies that, for times *t*≥*γ*^−1^, the BD model (1.6)–(1.7) is given by the BD model (6.1) shifted by time t*=3/(2*γ*). In particular, we will also add or subtract t* from the time variable whenever we switch between BD models.

**Figure 6.**
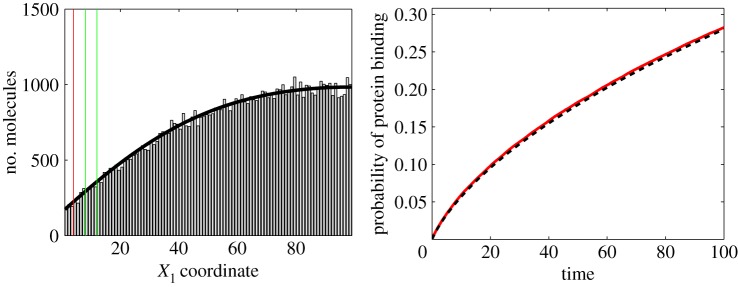
(*a*) Distribution of positions along the *X*_1_-axis at time *t*=1 computed by the multi-scale model described in §6 (grey histogram). The black solid line is the solution of the limiting PDE model (6.2) and (6.3). Vertical lines denote interfaces *I*, *I*_2_ and *I*_3_. (*b*) Probability that the protein is bound to a receptor as a function of time *t* computed by the multi-scale model (solid line) and the PDE model (6.2) and (6.3) (dashed line). Parameters used: *D*=10, *γ*=10^2^, *μ*=10^3^, *K*=1, *R*=1, *L*_1_=10^2^, *h*=4, *h*_2_=8, *h*_3_=12 and *h*_4_=3. (Online version in colour.)

In figure 6, we present illustrative results computed by averaging over 10^5^ realizations. Initial positions of the protein molecule are uniformly distributed along the *X*_1_-axis. The histogram of positions (along the *X*_1_-axis) at time *t*=1 is plotted in figure 6*a*. Interfaces *I*, *I*_2_ and *I*_3_ are also shown in this plot. As we used a very simple model of the protein binding, we can compare it with the mean-field limit given by the solution of the PDE

6.2 $$\frac{\partial \varrho }{\partial t}(x_{1},t)=D\frac{\partial^{2}\varrho }{\partial x_{1}^{2}}(x_{1},t),\;x_{1}\in [0,L_{1}],\;t\geq 0,$$

with boundary conditions [20]

6.3 $$D\frac{\partial \varrho }{\partial x_{1}}(0,t)=K\varrho (0,t)\;\mathrm{and}\;D\frac{\partial \varrho }{\partial x_{1}}(L_{1},t)=0,\;\text{where}\;P=\frac{K\sqrt{2\pi }}{\sqrt{D\gamma }}.$$

The solution of (6.2) and (6.3) with uniform initial condition *ϱ*(*x*_1_,0)≡const. is given by the black solid line in figure 6*a*. As we only visualize the distribution along the *X*_1_-axis in figure 6*a*, we can further decrease the computational cost by truncating the simulation domain in the *x*_2_ and *x*_3_ directions to the region close to the protein molecule. That is, we only simulate small particles in the subdomain [0,*h*]×[*X*_2_(*t*′)−*h*_4_,*X*_2_(*t*′)+*h*_4_]×[*X*_3_(*t*′)−*h*_4_,*X*_3_(*t*′)+*h*_4_], where *t*′ is the time when the protein molecule enters *Ω*_D_∪*Ω*_*C*1_. This subdomain (moving window) is shifted accordingly whenever *X*_2_(*t*) or *X*_3_(*t*) approach its boundary.

The probability that the protein is adsorbed to the surface is given as a function of time in figure 6*b*. It again compares well with the results obtained by the limiting PDE system (6.2) and (6.3).

## 7. Discussion

In this paper, a multi-scale approach that uses MD simulations in a part of the computational domain and BD simulations in the rest of the domain has been presented and analysed. The ultimate goal of this research is to use MD to help parametrize BD models of intracellular processes. One application area is modelling proteins in an aquatic environment, which is useful for understanding protein binding to receptors (surfaces) as shown in §6.

The main idea of the presented coupling of MD and BD models is based on using equations (4.1) and (5.1) and estimating drift and diffusion coefficients for velocities of molecules which cross the interface *I*. This coupling uses the same time step for the BD model (1.6)–(1.7) as for the MD model. In §6, it was shown that this is not a limiting step of this approach, because the BD model (1.6)–(1.7) is only needed in a small part of the domain next to *Ω*_D_. Then the coarser BD model (6.1) with larger time step can be used in the rest of the simulation domain, using a suitable overlap region. Another overlap region could be used to couple BD simulations with mean-field PDE-based models [11]. Then multi-scale models which couple BD (of point particles) with coarser reaction–diffusion approaches would be capable of further increasing time scales and space scales of simulations [10,11].

MD models considered in this paper are relatively simple and analytically tractable, describing water molecules as point particles. An important generalization is to consider more complicated MD models of water molecules [23]. For example, Rahman & Stillinger [24] model water molecules as rigid asymmetric rotors. That is, each water molecule is described by six coordinates: the position of its centre of mass and three angles describing molecule orientation. The energy of water solution is given as the sum of kinetic energies (for translation and rotation) and the intermolecular potential which is assumed to be pairwise additive and can be given in several different ways, i.e. the heat bath is given by its Hamiltonian [23–25]. I am currently investigating MD models based on Hamiltonian dynamics, with the aim of designing and analysing multi-scale algorithms similar to the algorithm [M1]–[M8] from this paper. The ultimate goal of this research is to design BD models of intracellular processes which make use of modern MD simulations [26,27] in small regions of intracellular space where a BD description is not available or applicable (and an MD level of detail is required). Results will be reported in a future publication.

## References

[1] Flegg M Rüdiger S Erban R 2013 Diffusive spatio-temporal noise in a first-passage time model for intracellular calcium release. J. Chem. Phys. 138, 154103 (doi:10.1063/1.4796417) 23614408 10.1063/1.4796417

[2] Takahashi K Tanase-Nicola S ten Wolde P 2010 Spatio-temporal correlations can drastically change the response of a MAPK pathway. Proc. Natl Acad. Sci. USA 107, 19820–19825 (doi:10.1073/pnas.0906885107) 20133748 10.1073/pnas.0906885107 PMC2811204

[3] Lipkow K Andrews S Bray D 2005 Simulated diffusion of phosphorylated CheY through the cytoplasm of?. Escherichia coli. 187, 45–53 (doi:10.1128/JB.187.1.45-53.2005) 10.1128/JB.187.1.45-53.2005 PMC538814 15601687

[4] Erban R Chapman SJ Maini P 2007 A practical guide to stochastic simulations of reaction–diffusion processes. (http://arxiv.org/abs/0704.1908) 10.1088/1478-3975/4/1/003 17406082

[5] Andrews S Bray D 2004 Stochastic simulation of chemical reactions with spatial resolution and single molecule detail. Phys. Biol. 1, 137–151 (doi:10.1088/1478-3967/1/3/001) 16204833 10.1088/1478-3967/1/3/001

[6] Stiles J Bartol T 2001 Monte Carlo methods for simulating realistic synaptic microphysiology using MCell. In Computational neuroscience: realistic modeling for experimentalists (ed. Schutter E), pp. 87–127 Boca Raton, FL: CRC Press

[7] van Zon J ten Wolde P 2005 Green's-function reaction dynamics: a particle-based approach for simulating biochemical networks in time and space. J. Chem. Phys. 123, 234910 (doi:10.1063/1.2137716) 16392952 10.1063/1.2137716

[8] Opplestrup T Bulatov V Donev A Kalos M Gilmer G Sadigh B 2009 First-passage kinetic Monte Carlo method. Phys. Rev. E 80, 066701 (doi:10.1103/PhysRevE.80.066701) 10.1103/PhysRevE.80.066701 20365296

[9] Erban R Flegg M Papoian G 2014 Multiscale stochastic reaction–diffusion modelling: application to actin dynamics in filopodia. Bull. Math. Biol. 76 (doi:10.1007/s11538-013-9844-3) 10.1007/s11538-013-9844-3 23640574

[10] Flegg M Chapman J Erban R 2012 The two-regime method for optimizing stochastic reaction–diffusion simulations. J. R. Soc. Interface 9, 859–868 (doi:10.1098/rsif.2011.0574) 22012973 10.1098/rsif.2011.0574 PMC3306650

[11] Franz B Flegg M Chapman J Erban R 2013 Multiscale reaction–diffusion algorithms: PDE-assisted Brownian dynamics. SIAM J. Appl. Math. 73, 1224–1247 (doi:10.1137/120882469)

[12] Lipkova J Zygalakis K Chapman J Erban R 2011 Analysis of Brownian dynamics simulations of reversible bimolecular reactions. SIAM J. Appl. Math. 71, 714–730 (doi:10.1137/100794213)

[13] Smoluchowski M 1917 Versuch einer mathematischen Theorie der Koagulationskinetik kolloider Lösungen. Z. Phys. Chem. 92, 129–168

[14] Erban R Chapman SJ 2009 Stochastic modelling of reaction–diffusion processes: algorithms for bimolecular reactions. Phys. Biol. 6, 046001 (doi:10.1088/1478-3975/6/4/046001) 19700812 10.1088/1478-3975/6/4/046001

[15] Holley R 1971 The motion of a heavy particle in an infinite one dimensional gas of hard spheres. Z. Wahrscheinlichkeitstheorie Verwandte Geb. 17, 181–219 (doi:10.1007/BF00536757)

[16] Dürr D Goldstein S Lebowitz J 1981 A mechanical model of Brownian motion. Commun. Math. Phys. 78, 507–530 (doi:10.1007/BF02046762)

[17] Dunkel J Hänggi P 2006 Relativistic Brownian motion: from a microscopic binary collision model to the Langevin equation. Phys. Rev. E 74, 051106 (doi:10.1103/PhysRevE.74.051106) 10.1103/PhysRevE.74.051106 17279876

[18] Robert C 1995 Simulation of truncated normal variables. Stat. Comput. 5, 121–125 (doi:10.1007/BF00143942)

[19] Hagan P Doering C Levermore C 1989 Mean exit times for particles driven by weakly colored noise. SIAM J. Appl. Math. 49, 1480–1513 (doi:10.1137/0149090)

[20] Erban R Chapman SJ 2007 Reactive boundary conditions for stochastic simulations of reaction–diffusion processes. Phys. Biol. 4, 16–28 (doi:10.1088/1478-3975/4/1/003) 17406082 10.1088/1478-3975/4/1/003

[21] Dror R Pan A Arlow D Borhani D Maragakis P Shan Y Xu H Shaw D 2011 Pathway and mechanism of drug binding to G-protein-coupled receptors. Proc. Natl Acad. Sci. USA 108, 13118–13123 (doi:10.1073/pnas.1104614108) 21778406 10.1073/pnas.1104614108 PMC3156183

[22] Vilaseca P Dawson K Franzese G 2013 Understanding and modulating the competitive surface-adsorption of proteins through coarse-grained molecular dynamics simulations. Soft Matter 9, 6978–6985 (doi:10.1039/c3sm50220a)

[23] Huggins D 2012 Correlations in liquid water for the TIP3P-Ewald, TIP4P-2005, TIP5P-Ewald, and SWM4-NDP models. J. Chem. Phys. 136, 064518 (doi:10.1063/1.3683447) 22360206 10.1063/1.3683447 PMC4766739

[24] Rahman F Stillinger F 1971 Molecular dynamics study of liquid water. J. Chem. Phys. 55, 3336–3359 (doi:10.1063/1.1676585)

[25] Mark P Nilsson L 2001 Structure and dynamics of the TIP3P, SPC, and SPC/E water models at 298 K. J. Phys. Chem. A 105, 9954–9960 (doi:10.1021/jp003020w)

[26] Merz K 2010 Limits of free energy computation for protein–ligand interactions. J. Chem. Theory Comput. 6, 1769–1776 (doi:10.1021/ct100102q) 10.1021/ct100102q PMC2866028 20467461

[27] Deng Y Roux B 2009 Computations of standard binding free energies with molecular dynamics simulations. J. Phys. Chem. B 113, 2234–2246 (doi:10.1021/jp807701h) 19146384 10.1021/jp807701h PMC3837708

